# Comparative assessment of respiratory‐induced tumor motion using fluoroscopy and four‐dimensional CT of solitary pulmonary lesions

**DOI:** 10.1002/acm2.70908

**Published:** 2026-11-14

**Authors:** Masato Kato, Takahiro Kato, Yuki Narita, Masao Murakami

**Affiliations:** ^1^ Department of Radiation Physics and Technology Southern Tohoku Proton Therapy Center Koriyama Fukushima Japan; ^2^ Department of Radiological Sciences, School of Health Sciences Fukushima Medical University Fukushima Japan; ^3^ Department of Radiation Oncology Southern Tohoku Proton Therapy Center Koriyama Fukushima Japan

**Keywords:** fluoroscopy, respiratory, tumor motion, 4DCT

## Abstract

**Background:**

Four‐dimensional computed tomography (4DCT) is widely used to quantify respiratory‐induced tumor motion in thoracic radiotherapy. However, 4DCT captures discretized respiratory phases and assumes stable breathing, potentially introducing reconstruction‐related uncertainties. Fluoroscopy enables real‐time observation across multiple breathing cycles, yet direct large‐scale comparisons between fluoroscopy‐ and 4DCT‐derived motion measurements remain scarce.

**Purpose:**

To directly compare respiratory‐induced tumor motion measured by fluoroscopy and 4DCT in patients with solitary pulmonary lesions undergoing proton therapy planning under free breathing without abdominal compression.

**Methods:**

This retrospective study included 191 patients with solitary pulmonary lesions who underwent sequential fluoroscopy and 4DCT under free‐breathing conditions as part of proton therapy planning. Tumor motion was evaluated in the craniocaudal direction and stratified by lung lobe. Furthermore, diaphragmatic motion was analyzed in 131 cases. Spearman's rank correlation coefficient was used to assess correlations between motions measured by the two modalities. Measurement differences were defined as ΔD (4DCT minus fluoroscopy) and analyzed according to fluoroscopy‐measured respiratory displacement. For subgroup analyses, cases were categorized into < 5 mm, ≥ 5 to ≤ 10 mm, and > 10 mm motion groups based on fluoroscopy‐measured respiratory displacement. For Bland–Altman analyses, motion magnitude was defined as the mean of the fluoroscopy‐ and 4DCT‐derived respiratory displacement measurements, and intermodality agreement was assessed within each motion category.

**Results:**

The correlation coefficients for tumor motion in the upper‐, middle‐, and lower‐lobes were 0.61 (*p* < 0.01), 0.76 (*p* < 0.01), and 0.55 (*p* < 0.01), respectively. The corresponding correlation coefficients for diaphragmatic motion were lower: 0.40 (*p* < 0.01) on the left and 0.38 (*p* < 0.01) on the right. The median |ΔD| values (interquartile range) were 1.3 mm (0.7–1.9 mm), 2.4 mm (1.0–4.1 mm), and 4.7 mm (2.6–6.8 mm) for the < 5 mm, ≥ 5 to ≤ 10 mm, and > 10 mm groups, respectively. Overall, the mean difference between 4DCT and fluoroscopy was small (−0.2 mm; 95% confidence interval, −0.9 to 0.6 mm), with 95% limits of agreement of −10.2 to 9.9 mm. The limits of agreement widened with increasing respiratory motion.

**Conclusions:**

In this single‐institution study of patients undergoing proton therapy planning, respiratory motion measurements derived from fluoroscopy and 4DCT showed larger absolute differences for lower‐lobe tumors (median |ΔD| = 4.20 mm) than for upper‐ and middle‐lobe tumors (1.70 and 1.90 mm, respectively). These findings indicate that respiratory motion estimated by 4DCT alone should be interpreted with caution in such cases. When feasible and when the lesion is fluoroscopically visible, fluoroscopic assessment may serve as a useful consistency check to complement 4DCT‐based motion evaluation.

## 1 INTRODUCTION

Respiratory motion management is an important consideration in external beam radiotherapy for tumors in organs such as the lungs and liver, where respiratory‐induced tumor motion can affect treatment delivery. The American Association of Physicists in Medicine Task Group 76 (AAPM TG‐76) recommends considering respiratory motion management when tumor motion exceeds approximately 5 mm, provided that it is expected to reduce the dose to normal tissues and can be appropriately implemented for the patient.^1^ Respiratory motion is an important challenge in proton therapy because changes in radiological path length can affect the delivered dose, while motion–delivery interplay can further alter dose distributions in scanned beam therapy.^2^ Accurate assessment of respiratory‐induced tumor motion is therefore important for determining whether motion management is warranted and for selecting an appropriate strategy. Common motion‐management strategies include respiratory gating, real‐time tumor tracking, breath‐hold techniques, and abdominal compression.^3^ Although abdominal compression may reduce respiratory motion, its feasibility and effects on patient anatomy must be considered,^4^ particularly in proton therapy, where anatomical changes can alter the water‐equivalent path length and increase uncertainty in proton range. Respiratory motion in lung lesions tends to be greater in the lower lobes than in the upper lobes, although interpatient variability makes it difficult to predict motion magnitude from tumor location alone.^5^, ^6^ Patient‐specific assessment over multiple breathing cycles is therefore important for selecting appropriate motion‐management strategies and determining parameters such as gating thresholds in gated irradiation.

Four‐dimensional computed tomography (4DCT) is widely used for respiratory motion assessment because it provides four‐dimensional information and good target visibility. However, 4DCT assumes relatively regular breathing during image acquisition, and irregular breathing or prolonged respiratory cycles can cause motion‐related artifacts.^7^ Although 4DCT reconstruction incorporates data acquired over multiple respiratory cycles, the reconstructed phase images represent composite respiratory states rather than the sequential variability of individual cycles. Fluoroscopy, in contrast, enables real‐time observation of tumor motion over multiple breathing cycles and may therefore provide complementary information to 4DCT.

Despite these complementary characteristics, direct comparisons of respiratory motion measured by fluoroscopy and 4DCT remain limited. To address this gap, we retrospectively compared respiratory motion measurements obtained using both modalities on the same day under free‐breathing conditions without abdominal compression in a large cohort of patients with solitary pulmonary lesions undergoing proton therapy planning.

## 2 MATERIALS AND METHODS

### 2.1 Patient background

This study included 191 patients with solitary pulmonary lesions who underwent sequential fluoroscopic examination and 4DCT for proton therapy planning at our institution. Lesions were distributed across the lung lobes: upper‐lobe, 72 cases; middle‐lobe, 48 cases; and lower‐lobe, 71 cases. Diaphragmatic motion was analyzed in 131 cases in which the diaphragm was included in the 4DCT scan range (left, 52 cases; right, 79 cases). The mean patient age was 71.7 ± 10.1 years (range 41–91 years; median 73 years). This retrospective study was approved by the institutional review board of our institution (approval number: 783).

### 2.2 Imaging procedure

Figure 1 shows the fluoroscopic and CT images from this study. The imaging workflow involved sequential acquisition using two modalities. First, continuous fluoroscopic imaging was performed in two orthogonal directions (anterior‐posterior and lateral) in the fluoroscopy room prior to CT simulation. Second, patients were transferred to the adjacent CT room, where a respiratory‐gated planning CT was acquired, followed immediately by a 4DCT scan. In both modalities, respiratory displacement was defined as the positional difference between end‐inspiration and end‐expiration. Preliminary verification using a dynamic phantom was performed prior to the study to confirm measurement consistency between fluoroscopy and 4DCT. Preliminary phantom validation demonstrated agreement between fluoroscopy and 4DCT measurements within approximately 1 mm. All examinations were conducted under free‐breathing conditions. At our institution, respiratory‐gated irradiation is employed for respiratory motion management. Given the uncertainty associated with proton beam range and the need to maintain reproducibility of body contour changes, abdominal compression is not routinely used. Each imaging modality and acquisition protocol is described in detail.

**FIGURE 1:**
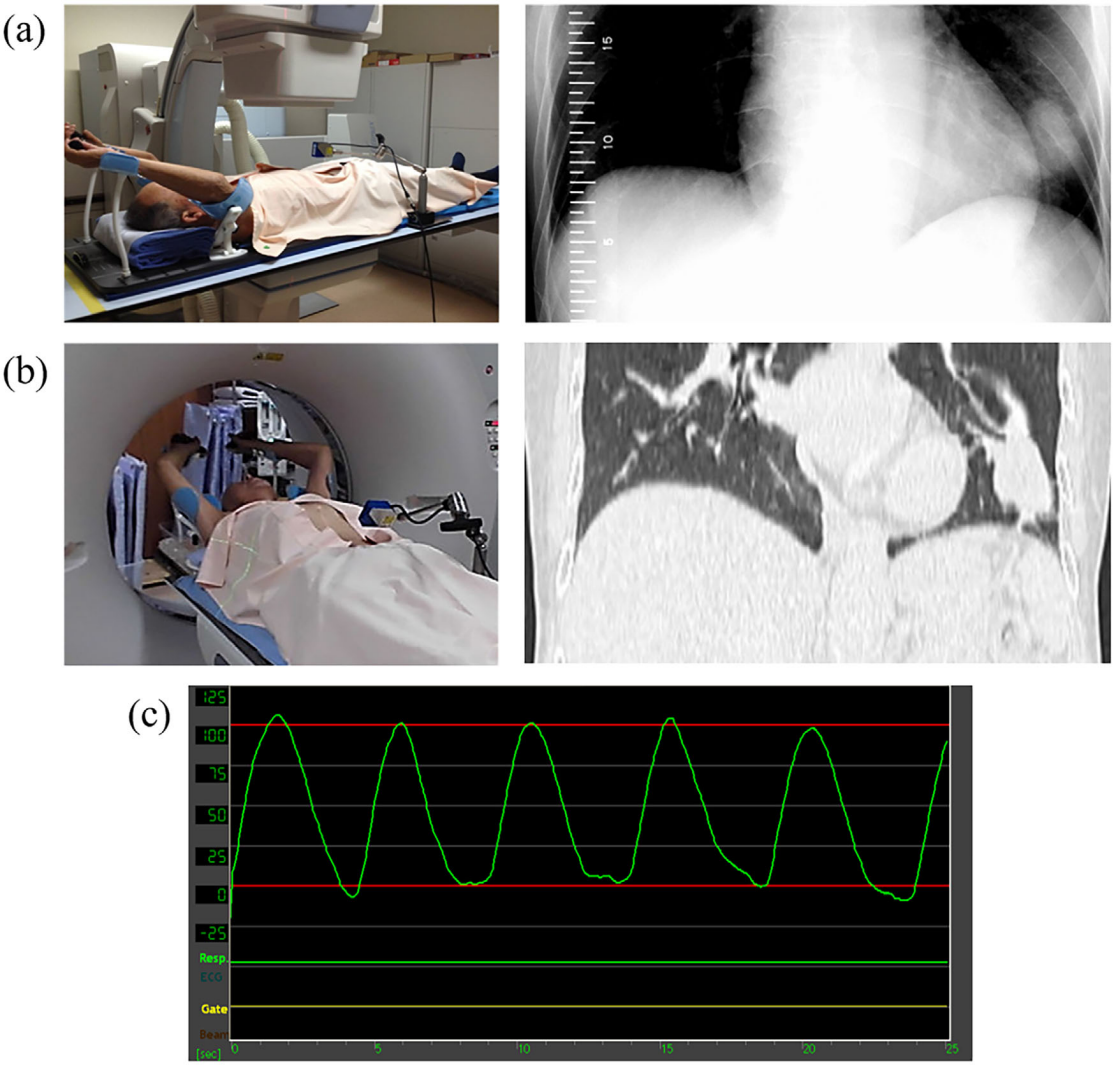
Overviews of fluoroscopic examination (a), CT examination (b), and representative surface motion traces (c).

#### 2.2.1 Fluoroscopy

Fluoroscopic imaging was performed using a multifunctional digital X‐ray fluoroscopy system (ULTIMAX 80; Canon Medical Systems, Tochigi, Japan). Patients were positioned supine with both arms raised above the head, and fluoroscopic images were acquired in two orthogonal directions: anterior‐posterior and lateral views. A respiratory monitoring system (AZ‐733 V; Anzai Medical Co., Ltd., Tokyo, Japan) was used to monitor abdominal wall motion. This respiratory monitoring system is a markerless infrared optical tracking system that generates respiratory waveforms based on abdominal surface motion. Continuous fluoroscopic acquisition was initiated after visual confirmation of a regular respiratory pattern by the operator without abrupt changes in respiratory amplitude or cycle, coughing, or excessively deep or shallow breathing. The imaging parameters were tube voltage (80 kV), tube current (200 mA), and frame rate (6 frames per second). The acquisition duration was adjusted according to the patient's breathing pattern and typically covered 2 respiratory cycles for each projection. Respiratory motion was quantified by tracking margins of the tumor. Diaphragmatic motion was also evaluated, as the diaphragm is generally considered to exhibit among the greatest respiratory excursions in the human body,^8^ and comparison of its motion may better reveal differences between the two imaging modalities. In fluoroscopic imaging, craniocaudal (CC) displacement was evaluated using anterior–posterior and lateral projection images. As fluoroscopic images are subject to geometric magnification, pixel size was calibrated using a reference object of known dimensions visible in the detector field (Figure 2a). For the tumor and diaphragm, respiratory displacement was measured in both anterior‐posterior and lateral views as the distance between the end‐expiratory and end‐inspiratory positions across all phases. The measured displacement was corrected for geometric magnification using the source‐to‐image distance at image acquisition and the source‐to‐object distance, which was estimated from tumor depth on diagnostic or treatment planning CT. The final displacement value used for analysis was taken as the maximum value over the several respiratory cycles.

**FIGURE 2:**
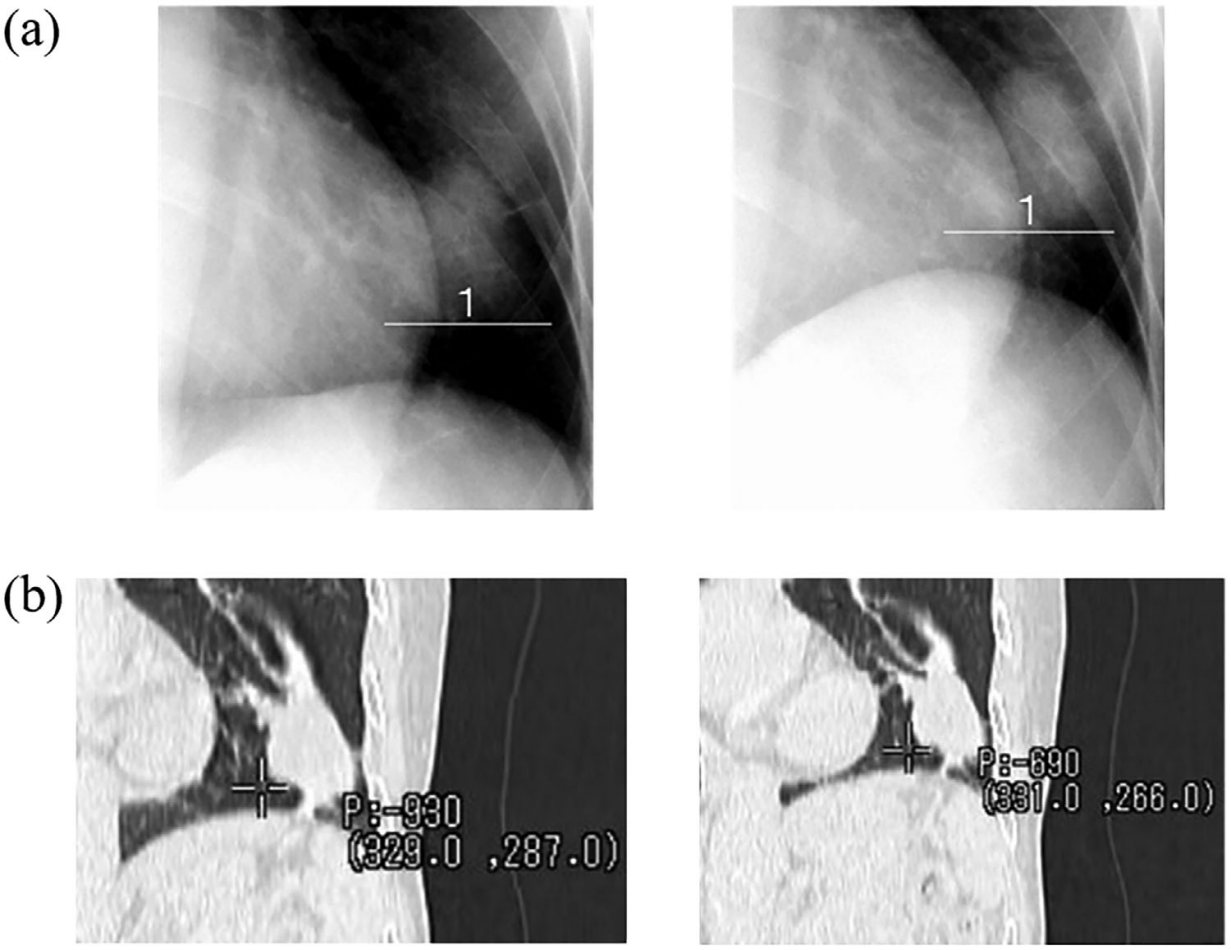
Representative fluoroscopic anterior–posterior images (a) and corresponding 4DCT coronal images (b) at the inspiratory phase (left panels) and expiratory phase (right panels). The images are screenshots of the imaging console obtained during measurement of the inferior tumor margin. Horizontal bars and crosses indicate the measured margin used to determine craniocaudal displacement. Additional numerical values, including coordinates and measurement parameters, are automatically displayed on the console. Differences in image appearance between fluoroscopy and 4DCT may make direct identification of identical anatomical landmarks challenging.

#### 2.2.2 4DCT

4DCT was performed using a 16‐slice CT scanner (Aquilion LB; Canon Medical Systems, Tochigi, Japan). Patient positioning was similar to that used for fluoroscopic imaging, and using the same respiratory monitoring system as used for fluoroscopy, abdominal surface motion was measured within the same region of interest. The acquisition parameters were tube voltage (120 kV), tube current (350 mA), gantry rotation time (0.75 s), slice thickness (2 mm), and field of view (550 mm). The helical pitch was determined on the CT console based on the patient's respiratory cycle measured by the respiratory monitoring system. Raw 4DCT data were sorted into 10 respiratory phases by phase binning based on the gating signal. Pixel coordinates corresponding to the same anatomical landmarks used for fluoroscopic analysis were identified on coronal and sagittal CT images at each respiratory phase (Figure 2b). Respiratory motion was calculated as the distance between the coordinates at end‐inspiration and end‐expiration. The in‐plane pixel size was 0.91 mm. Window level and width settings were fixed for all analyses to ensure consistency across respiratory phases. For 4DCT, the larger of the displacement values measured in the coronal and sagittal planes was adopted as the final displacement value. Furthermore, cases with extremely irregular respiration and obvious reconstruction failure during 4DCT acquisition were excluded from the evaluation.

### 2.3 Analysis

Respiratory‐induced motion is known to be most pronounced in the CC direction compared with anterior–posterior and left–right directions.^5^ As the primary objective of this study was to compare measurement methodologies, analysis focused on CC motion, which is the dominant component of respiratory displacement. Accordingly, respiratory motions of the tumor and ipsilateral diaphragm were evaluated in the CC direction, using tumor margin and the most cranial diaphragmatic point.

The association between respiratory motion measured by fluoroscopy and 4DCT was assessed using Spearman's rank correlation coefficient. Tumors were stratified by anatomical location (upper, middle, and lower‐lobes), and diaphragmatic motion was analyzed separately for the left and right sides. Measurement differences between the two modalities were defined as follows:

$$\begin{array}{cc} \Delta D & =\left(4\mathrm{DCT}\;\mathrm{measured}\;\mathrm{value}\right)\\ & -\left(\mathrm{fluoroscopy}\;\mathrm{measured}\;\mathrm{value}\right) \end{array}$$

The magnitude of measurement differences, expressed as |ΔD|, was compared across tumor locations (upper‐, middle‐, and lower‐lobes) and between the left and right diaphragms using the Mann–Whitney U test. Tumors were similarly stratified according to respiratory displacement measured by fluoroscopy (< 5 mm, ≥ 5 to ≤ 10 mm, and > 10 mm) for additional analysis. Furthermore, Bland–Altman analyses were performed to evaluate agreement between respiratory motion measurements obtained using fluoroscopy and 4DCT. Tumors were stratified according to the mean of the respiratory displacements measured by fluoroscopy and 4DCT (< 5 mm, ≥ 5 to ≤ 10 mm, and > 10 mm). The mean difference and 95% limits of agreement (LoA) between the two measurement methods were calculated.

## 3 RESULTS

Figure 3 shows scatter plots illustrating the relationship between respiratory motion measured by fluoroscopy and 4DCT for tumors and the diaphragm. The correlation coefficients for tumor motion in the upper‐, middle‐, and lower‐lobes were 0.61 (*p* < 0.01), 0.76 (*p* < 0.01), and 0.55 (*p* < 0.01), respectively. In contrast, the corresponding correlation coefficients for diaphragmatic motion were lower (0.40 [*p* < 0.01] on the left and 0.38 [*p* < 0.01] on the right), indicating a consistently weaker correlation for the diaphragm.

**FIGURE 3:**
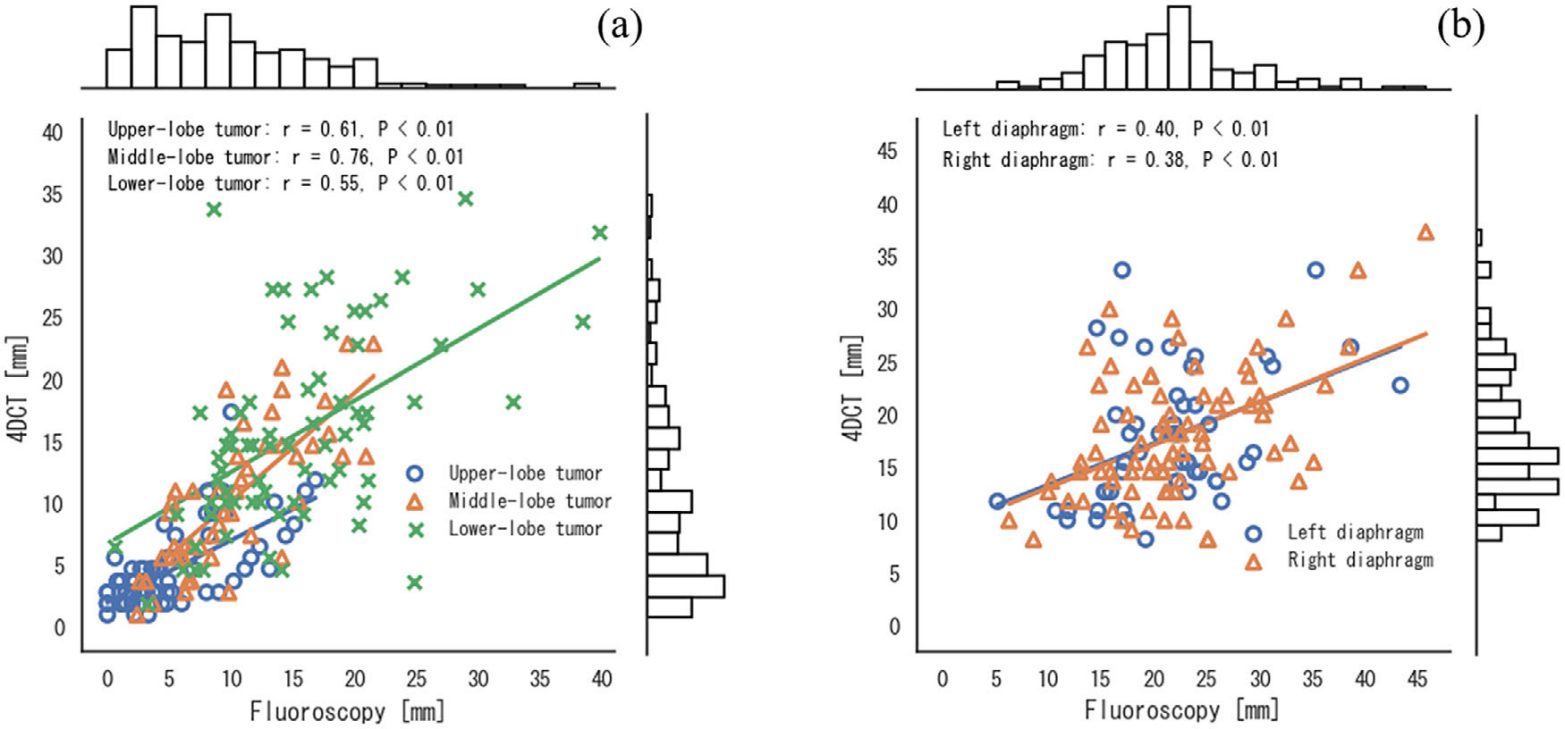
Correlation plots comparing respiratory motions measured by fluoroscopy and 4DCT for the tumor (a) and diaphragm (b).

Figure 4a presents box‐and‐whisker plots of |ΔD| for tumors stratified by lung lobe (upper, middle, and lower) and for the left and right diaphragm. The median (interquartile range) of |ΔD| for tumors was 1.70 mm (0.90–2.80 mm) in the upper‐lobe, 1.90 mm (0.98–4.15 mm) in the middle‐lobe, and 4.20 mm (2.45–6.65 mm) in the lower‐lobe. The magnitude of |ΔD| was significantly larger for lower‐lobe lesions than for upper‐ and middle‐lobe lesions (*p* < 0.001). The median (interquartile range) of |ΔD| for the diaphragm was 5.05 mm (2.28–9.40 mm) on the left and 5.20 mm (2.90–8.65 mm) on the right, with no significant difference between sides (*p* > 0.05). Among tumors, the number of cases with a measurement difference of > 5 mm was: 11 cases (15.3%) in the upper‐lobe, 10 cases (20.8%) in the middle‐lobe, and 28 cases (39.4%) in the lower‐lobe. Measurement differences of > 10 mm were observed in eleven cases, all of which were located in the lower‐lobe (15.5%); no such cases were identified in the upper‐ and middle‐lobes. These findings indicate that the largest discrepancies between fluoroscopic and 4DCT measurements occurred for lower‐lobe tumors. Figure 4b shows box plots of |ΔD| stratified according to respiratory displacement measured by fluoroscopy (< 5 mm, ≥ 5 to ≤ 10 mm, and > 10 mm). The median |ΔD| values (interquartile range) was 1.3 mm (0.7–1.9 mm), 2.4 mm (1.0–4.1 mm), and 4.7 mm (2.6–6.8 mm) for the < 5 mm, ≥ 5 to ≤ 10 mm, and > 10 mm groups, respectively. Cases with a measurement difference exceeding 5 mm were observed in 1 of 55 cases (1.8%), 9 of 52 cases (17.3%), and 39 of 84 cases (46.4%) in the < 5 mm, ≥ 5 to ≤ 10 mm, and > 10 mm groups, respectively. Cases with a measurement difference exceeding 10 mm were observed in 0 cases, 1 case (1.9%), and 10 cases (11.9%), respectively.

**FIGURE 4:**
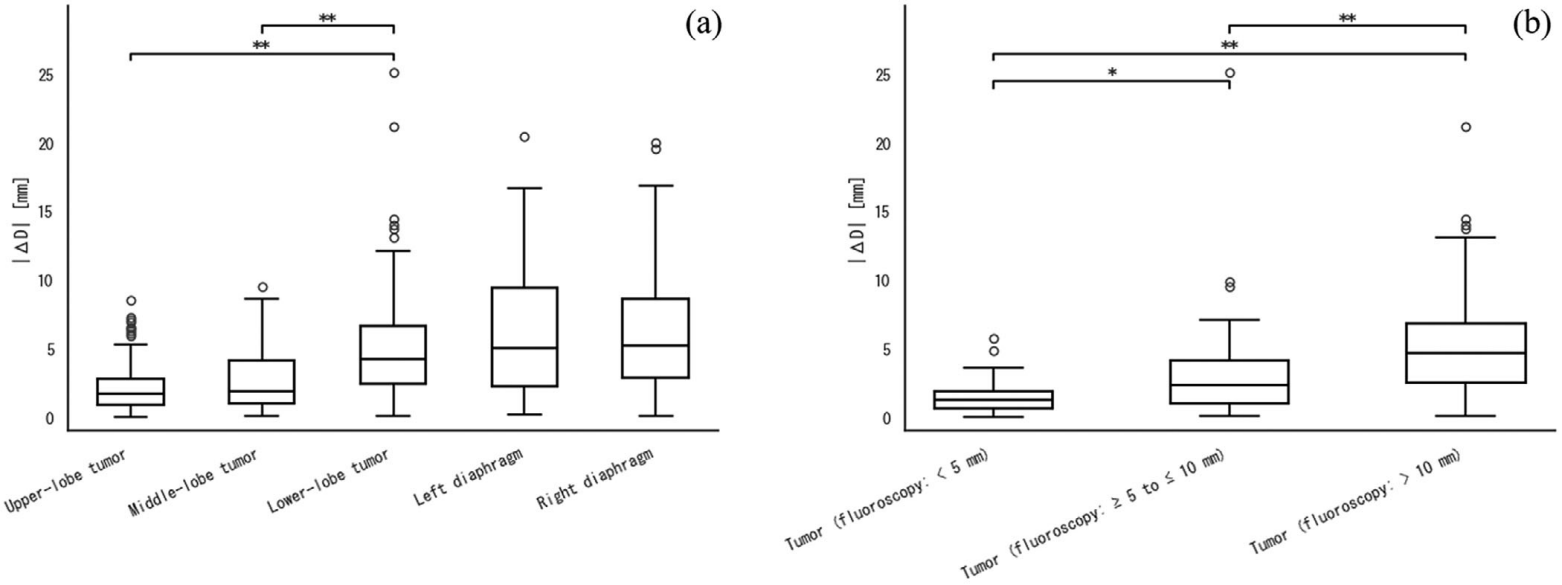
Box‐and‐whisker plots of |ΔD| for tumors in the upper‐, middle‐, and lower‐lung lobes and for the diaphragm (a), and for tumors stratified into three groups according to respiratory displacement measured by fluoroscopy: < 5 mm, ≥ 5 to ≤ 10 mm, and > 10 mm (b). Significant differences are indicated by an asterisk (*p < 0.05, **p < 0.01).

Figure 5 shows the Bland–Altman plots illustrating measurement differences between fluoroscopy and 4DCT. For tumor motion across all cases, the mean difference between 4DCT and fluoroscopy was −0.18 mm (95% confidence interval, −0.91 to 0.55 mm), with 95% LoA of −10.2 to 9.9 mm. When stratified according to the mean of the fluoroscopy‐ and 4DCT‐derived measurements, the 95% LoA were −3.7 to 4.1 mm, −8.8 to 5.8 mm, and −13.2 to 13.8 mm for the < 5 mm, ≥5 to ≤10 mm, and > 10 mm groups, respectively. The LoA were wider in groups with greater respiratory motion. For diaphragmatic motion, the mean difference was −3.9 mm (95% confidence interval, −5.1 to −2.7 mm), with 95% LoA of −17.4 to 9.6 mm.

**FIGURE 5:**
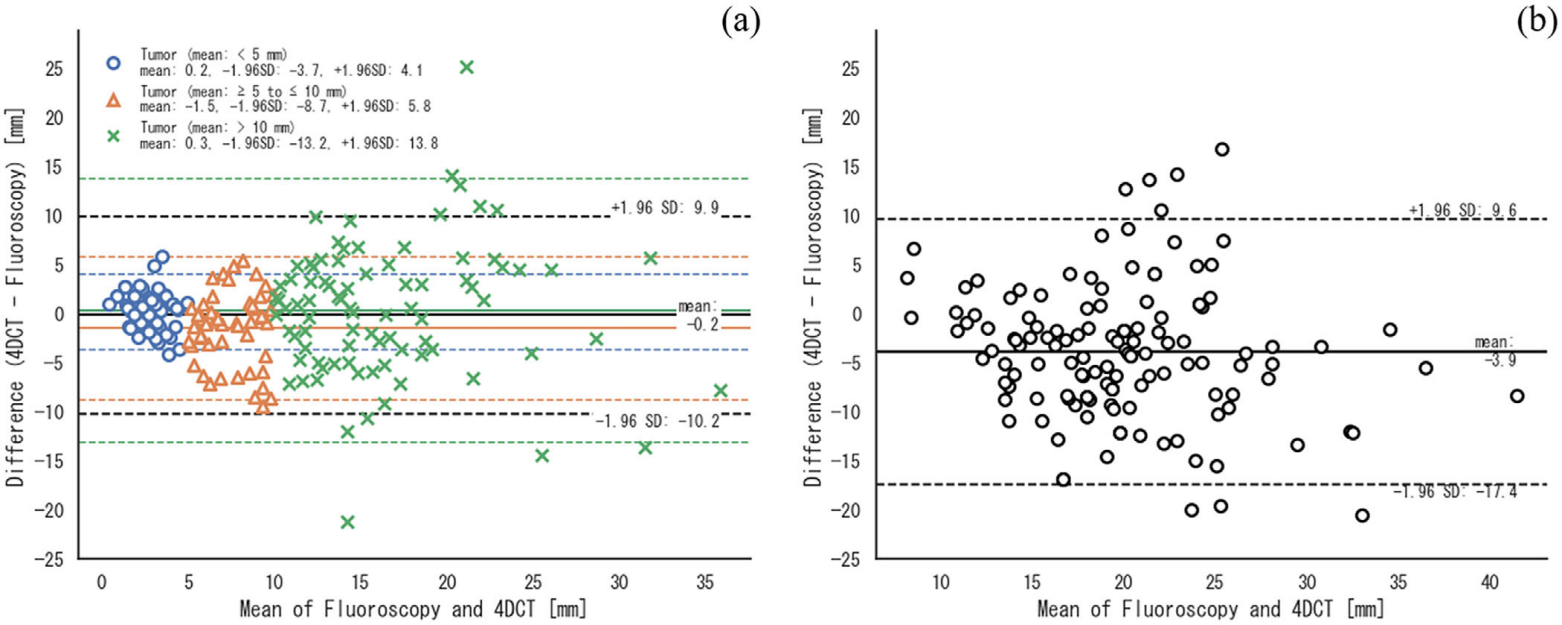
Bland–Altman plots of respiratory displacement of tumors (a) and diaphragms (b) measured by fluoroscopy and 4DCT. The x‐axis shows the mean of the two methods, and the y‐axis shows the difference (4DCT − fluoroscopy). Tumors are stratified by the mean of the two methods (< 5 mm, ≥ 5 to ≤ 10 mm, > 10 mm). Solid and dashed lines indicate bias and 95% limits of agreement, respectively. The black line represents overall results.

## 4 DISCUSSION

The principal finding of the present study was that the agreement between respiratory motion measurements obtained using fluoroscopy and 4DCT depended strongly on the magnitude of respiratory motion. The 95% LoA for tumor motion overall became wider as respiratory displacement increased. Consistent with this finding, the absolute measurement difference between the two modalities was significantly greater for lower‐lobe tumors than for upper‐ and middle‐lobe tumors and increased progressively across the < 5 mm, ≥ 5 to ≤ 10 mm, and > 10 mm motion groups. Among tumors, the correlation between the two modalities was strongest for middle‐lobe lesions (*r* = 0.76) and weakest for lower‐lobe lesions (*r* = 0.55), whereas the correlations for diaphragmatic motion were lower (*r* = 0.40 on the left and 0.38 on the right). Importantly, the present study was not designed to determine which modality provides the more accurate measurement. Rather, the findings suggest that respiratory motion assessed at a single simulation session may not fully capture physiologic variability over time.

A methodological strength of the present study was the comparison of fluoroscopy and 4DCT within a large cohort using a standardized respiratory monitoring environment. AAPM TG‐76 recommends evaluating respiratory motion over multiple breathing cycles. Fluoroscopy allows direct real‐time observation of consecutive respiratory cycles, whereas 4DCT reconstructs discretized respiratory phases from data acquired over multiple cycles. Conventional fluoroscopic systems, however, are not routinely integrated with respiratory monitoring systems, which may limit the reliability of fluoroscopy‐based motion assessment for radiotherapy applications. In the present study, the same respiratory monitoring system was used during both fluoroscopy and 4DCT, and fluoroscopic acquisition was performed only after visual confirmation of a regular respiratory pattern. Furthermore, preliminary dynamic phantom measurements demonstrated agreement between the two modalities within approximately 1 mm. Thus, the larger discrepancies observed clinically, particularly for lesions with greater respiratory excursion, are unlikely to be explained solely by basic differences in measurement accuracy between the imaging systems. Differences in image appearance between fluoroscopy and 4DCT may make direct identification of identical anatomical landmarks challenging.

Several factors may contribute to the larger discrepancies observed with increasing respiratory motion. Respiratory amplitude, baseline position, and cycle‐to‐cycle variation can change over time even under free‐breathing conditions, and such physiologic variability may become more pronounced as respiratory excursion increases. Differences in the timing and nature of motion assessment between fluoroscopy and 4DCT may also contribute to the observed intermodality discrepancies. Respiratory phase images reconstructed by 4DCT represent composite respiratory states rather than the continuous trajectory of an individual respiratory cycle. Reconstruction artifacts are known to occur more frequently in anatomical regions with large respiratory motion, particularly near the diaphragm,^7^ and may become more pronounced as motion velocity increases. Baseline shifts and other respiratory irregularities may also affect 4DCT reconstruction. These factors may partly explain the large discrepancies observed for lower‐lobe tumors and the particularly weak agreement observed for diaphragmatic motion. However, the present study was not designed to determine which modality provides the more accurate measurement. The present findings should therefore not be interpreted as indicating an inherent inaccuracy of either modality, but rather as reflecting the potentially combined effects of respiratory variability, temporal sampling, and modality‐specific image acquisition and reconstruction.

The clinical relevance of these findings should be interpreted in the context of respiratory motion management. The patients in the present study were evaluated under free‐breathing conditions without abdominal compression. In many institutions, patients with larger respiratory motion may instead undergo breath‐hold, abdominal compression, respiratory‐gated irradiation, or real‐time tumor tracking. Therefore, the magnitude of the discrepancies observed in the large‐motion subgroup may not be directly applicable to workflows in which respiratory excursion is actively reduced. Nevertheless, these findings suggest that uncertainty in motion estimation may increase with respiratory excursion. Consequently, a large respiratory excursion identified during simulation may warrant particular attention not only because of the magnitude of motion itself but also because of potentially reduced measurement reproducibility. Although abdominal compression remains associated with considerations such as reproducibility of compression, changes in body contour, and deformation of internal anatomy,^4^ it is one approach to reducing respiratory motion and may potentially reduce uncertainty in respiratory motion assessment by limiting respiratory excursion. Respiratory‐gated irradiation, as used in the present study, has been widely adopted in particle therapy in certain regions and is routinely performed at our institution;^9^ therefore, abdominal compression was not incorporated into the present workflow. Nevertheless, further investigation and refinement of effective abdominal compression strategies in particle therapy may contribute to improving respiratory motion management.

Differences in estimated respiratory motion may also have dosimetric implications. In lung SBRT, steep dose gradients are intentionally generated around the target. Previous studies reported that the distance between the 100% and 50% isodose lines may be approximately 10 mm for small PTVs.^10^ Furthermore, motion simulation studies have demonstrated that target displacement of several millimeters can affect peripheral target dose, with substantially larger reductions as motion approaches 10 mm.^11^ In proton pencil beam scanning, a steep lateral penumbra can similarly occur at the field edge, with dose falloff over several millimeters depending on beam configuration and depth.^12^ In addition, in proton therapy, interplay between respiratory motion and beam delivery can alter dose distributions in scanned beam therapy, while changes in water‐equivalent path length may affect proton range.^2^ Respiratory motion can also alter the geometric position of the target and the water‐equivalent thickness along the beam path, potentially producing dosimetric effects beyond those attributable to geometric displacement alone.^13^ Although the present study did not directly evaluate dose distributions, the observed intermodality discrepancies may have implications for dosimetric assessment, particularly in cases with larger respiratory motion. Therefore, the potential impact of respiratory motion measurement uncertainty on internal margin determination, gating thresholds, and target coverage warrants further investigation. Reliance on a single respiratory motion estimate should be approached cautiously, as respiratory motion measurements may be subject to greater uncertainty as respiratory motion increases.

Several technical characteristics of the two modalities should also be considered when interpreting their agreement. Fluoroscopic tumor visibility may be reduced for ground‐glass opacity lesions or lesions overlapping complex anatomical structures. In some cases, pulmonary vessels or other anatomical structures moving synchronously with the tumor may serve as useful surrogate landmarks, as demonstrated in previous studies of markerless fluoroscopic tumor tracking.^14^ In addition, fluoroscopy provides two‐dimensional projection images, whereas 4DCT provides three‐dimensional volumetric information. For 4DCT, reconstruction performance may depend on the CT system and reconstruction strategy. Advances in CT technology may reduce these reconstruction‐related uncertainties. Previous studies demonstrated using respiratory waveform‐driven dynamic phantom experiments that a wide‐detector CT system reduced reconstruction errors compared with a 16‐detector‐row system.^15^ Amplitude‐based sorting has also been reported to reduce reconstruction artifacts compared with phase‐based sorting in patients with irregular respiration.^16^ Cine‐mode acquisition has been shown to be more robust than helical acquisition under unstable respiratory conditions.^17^ Respiratory coaching may further stabilize respiratory patterns, although its effect varies among patients and verbal instruction may occasionally increase abdominal wall motion.^18^ These modality‐specific characteristics emphasize that fluoroscopy and 4DCT provide complementary rather than interchangeable information on respiratory motion.

From a practical perspective, the present results support the value of complementary respiratory motion assessment when unstable respiratory motion is suspected. Because 4DCT provides essential three‐dimensional anatomical and respiratory information, it remains the primary imaging method for treatment planning and should not be replaced by fluoroscopy. However, fluoroscopy provides a different type of information by directly displaying respiratory motion over consecutive breathing cycles. When available and when the lesion can be adequately visualized, fluoroscopic assessment may serve as a consistency check for 4DCT‐derived motion. Although dedicated simulation‐room fluoroscopy may not be available at all institutions, most modern treatment systems provide on‐board imaging capable of fluoroscopic observation. Reassessment of respiratory motion during patient setup or treatment verification may provide an opportunity to confirm whether the respiratory motion observed at simulation remains representative during the treatment course. Such reassessment may be particularly useful when changes or instability in respiratory motion are suspected. Where clinically appropriate, this additional information may support reliable determination of internal margins, gating thresholds, and respiratory motion‐management strategies.

Several limitations of this study should be acknowledged. First, fluoroscopy and 4DCT were acquired sequentially in different rooms rather than simultaneously. Although both examinations were performed on the same day under free‐breathing conditions and respiratory stability was confirmed before fluoroscopic acquisition, changes in respiratory amplitude, baseline position, or breathing pattern between examinations cannot be excluded. The effect of such physiologic variability was assumed to be small based on previous reports;^19^, ^20^ however, its contribution to the observed measurement differences remains uncertain, particularly for lower‐lobe tumors. In addition, respiratory waveform data were not retained for quantitative comparison; therefore, the extent to which physiologic variability contributed to the observed discrepancies could not be determined. Second, fluoroscopy is inherently a two‐dimensional projection technique and cannot fully characterize out‐of‐plane motion, tumor deformation, or rotation. Tumor visibility may also affect measurement accuracy, particularly for lesions with low radiographic contrast, and lateral views may be degraded by patient thickness or overlap with cardiac and mediastinal structures. Third, the analysis was restricted to CC motion, which generally represents the dominant component of respiratory displacement, and therefore does not characterize three‐dimensional tumor motion. Fourth, this was a single‐institution study performed using a 16‐detector‐row CT scanner with phase‐based respiratory sorting. The magnitude of the observed discrepancies may therefore differ with newer CT systems, different acquisition modes such as helical or cine acquisition, alternative reconstruction or sorting methods, and different institutional motion‐management workflows. Finally, all patients were evaluated under free‐breathing conditions without abdominal compression, and the findings may not be directly generalizable to patients managed using abdominal compression. Future studies incorporating repeated motion measurements, quantitative respiratory waveform analysis, three‐dimensional motion assessment, and contemporary 4DCT techniques would help clarify the relative contributions of physiologic variability and imaging‐related uncertainty to the observed intermodality differences.

## 5 CONCLUSION

This large single‐institution study compared respiratory motion measured by fluoroscopy and 4DCT in patients with solitary pulmonary lesions under free‐breathing conditions without abdominal compression during proton therapy planning. Agreement between respiratory motion measurements obtained using fluoroscopy and 4DCT decreased with increasing respiratory motion, with lower correlations for lower‐lobe tumors and the diaphragm. The increasing intermodality variability with larger respiratory excursions indicates that a single measurement may not fully characterize respiratory motion in such cases. These findings highlight the importance of considering the reproducibility and uncertainty of respiratory motion assessment as respiratory motion increases. Fluoroscopy should not replace 4DCT for treatment planning; however, when feasible and when the lesion is adequately visualized, it may provide a useful complementary consistency check to support individualized respiratory motion management.

## AUTHORS CONTRIBUTION

**Masato Kato**: Conceptualization; investigation; methodology; formal analysis; writing—original draft. **Takahiro Kato**: Methodology; writing—review & editing. **Yuki Narita**: writing—review & editing. **Masao Murakami**: writing—review & editing.

## CONFLICT OF INTEREST STATEMENT

The authors declare that they have no competing financial or personal interests that may have influenced this study.

## ETHICS STATEMENT

This retrospective study was approved by the institutional review board of Southern Tohoku Proton Therapy Center (approval number: 783).

## Data Availability

The data that support the findings of this study are available from the corresponding author upon reasonable request.
